# Polygenic scores contribution to Parkinson’s disease comorbidities

**DOI:** 10.1093/braincomms/fcaf325

**Published:** 2025-08-29

**Authors:** Carlos F Hernández, Camilo Villaman, Cristian Tejos, Gabriela M Repetto, Costin Leu, Dennis Lal, Ignacio F Mata, Andrés D Klein, Eduardo Pérez-Palma

**Affiliations:** Universidad del Desarrollo, Centro de Genética y Genómica, Facultad de Medicina Clínica Alemana, Santiago 7610658, Chile; Universidad del Desarrollo, Centro de Genética y Genómica, Facultad de Medicina Clínica Alemana, Santiago 7610658, Chile; Department of Electrical Engineering, Pontificia Universidad Católica de Chile, Santiago 7820436, Chile; Biomedical Imaging Center, Pontificia Universidad Católica de Chile, Santiago 7820436, Chile; Millennium Institute for Intelligent Healthcare Engineering, Santiago 7820436, Chile; Universidad del Desarrollo, Centro de Genética y Genómica, Facultad de Medicina Clínica Alemana, Santiago 7610658, Chile; Center for Neurogenetics, The University of Texas Health Science Center at Houston, Houston, TX 77030, USA; Department of Clinical and Experimental Epilepsy, UCL Queen Square Institute of Neurology, University College London, London WC1N 3BG, UK; Center for Neurogenetics, The University of Texas Health Science Center at Houston, Houston, TX 77030, USA; Department of Neurology, The University of Texas Health Science Center at Houston, Houston, TX 77030, USA; Stanley Center for Psychiatric Research, Broad Institute of MIT and Harvard, Cambridge, MA 02142, USA; Cologne Center for Genomics (CCG), Medical Faculty of the University of Cologne, Köln 50923, Germany; Genomic Medicine Institute, Cleveland Clinic Research, Cleveland Clinic, Cleveland, OH 44195, USA; Universidad del Desarrollo, Centro de Genética y Genómica, Facultad de Medicina Clínica Alemana, Santiago 7610658, Chile; Universidad del Desarrollo, Centro de Genética y Genómica, Facultad de Medicina Clínica Alemana, Santiago 7610658, Chile

**Keywords:** polygenic scores, comorbidities, common variants, polygenic risk scores

## Abstract

Comorbidities are common in Parkinson’s disease and significantly impact the disease progression and management. While polygenic scores have been widely used to assess genetic risk for complex diseases, their role in comorbidity presentation in Parkinson’s disease remains unclear. This study investigates whether genetic predisposition to comorbidities, as measured by polygenic scores, differs between individuals with Parkinson’s disease and the general population and explores how genetic risk influences disease onset and sex-related differences. We analysed data from 4144 individuals with Parkinson’s disease and 370 480 individuals from the general population in the UK Biobank, focusing on four comorbidities with high-quality genome-wide association study data: Type 2 diabetes, major depressive disorder, migraine headaches and epilepsy. We first compared polygenic score distributions between individuals with Parkinson’s disease and the general population. While our findings indicate that comorbidities and polygenic risk scores do not significantly differ between individuals with Parkinson’s disease and the general population, we show an association with disease onset and sex-specific differences. Individuals with earlier disease onset (50–70 years old) had higher genetic risk for major depressive disorder (odds ratio: 2.19, *P*-value: 1.27 × 10⁻¹⁵) and epilepsy (odds ratio: 1.58, *P*-value: 0.00845). Additionally, a female participant with Parkinson’s disease exhibited higher genetic risk scores for major depressive disorder (odds ratio: 1.5, *P*-value: 0.0119) and migraine headaches (odds ratio: 2.1, *P*-value: 0.0155), while a male participant displayed higher genetic risk scores for Type 2 diabetes (odds ratio: 2.7, *P*-value: 2.11 × 10⁻¹⁷). Comorbidity-polygenic score did not differ between people with versus without Parkinson’s disease, yet within Parkinson’s disease, a higher genetic burden for specific comorbidities was linked to earlier onset and sex-specific presentation, implicating common variants as modifiers of clinical heterogeneity rather than the primary disease risk. These results enhance our understanding of the genetic influences shaping the broader clinical presentation of Parkinson’s disease and highlight the need for further research into the interplay between genetic risk factors, comorbidities and disease heterogeneity.

## Teaser

Hernández et al. reported that genetic predisposition to comorbidities does not significantly differ between individuals with Parkinson’s disease and the general population, suggesting independence from disease status. However, genetic risk influences disease onset and sex-specific differences, highlighting the role of polygenic scores in shaping Parkinson’s disease presentation and clinical heterogeneity.

## Graphical Abstract

**Graphical Abstract:**
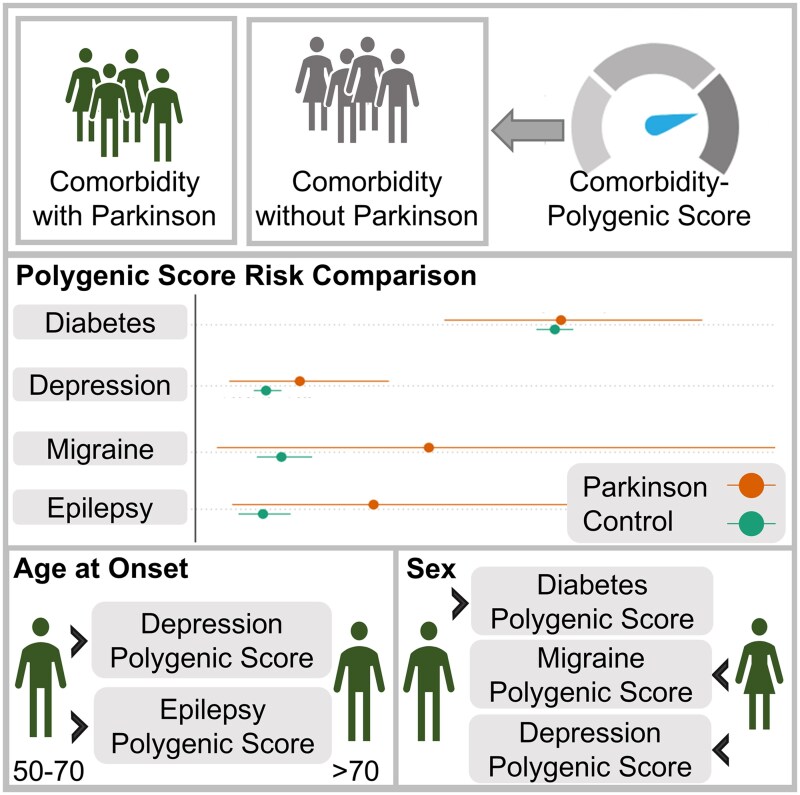

## Introduction

Parkinson’s disease (PD) is the second most common neurodegenerative disease after Alzheimer’s disease. It has a global incidence of 315 cases per 100 000 people annually, increasing to 1087 cases per 100 000 in people over the age 70.^1^ The majority of PD cases are sporadic, with no clear genetic cause, and have a multifactorial origin.^2^

Comorbidities are frequently observed in PD patients and impact their quality of life.^3^ The most common are Type 2 diabetes (T2D), depression, migraines, hypertension and cancer.^4,5^ Recently, other comorbidities have been described, such as epilepsy (EPI) and hearing loss.^6^ Comorbidities are typically associated with disease severity.^7^ For example, T2D, which is associated with increased PD risk, is also linked to faster motor progression and cognitive decline.^8,9^

Genetically, common genetic variation identified by genome-wide association studies (GWAS) has been extensively associated with PD presentation. To date, the largest PD GWAS has associated 90 independent loci with PD risk in European populations.^10^ GWAS can also be used to determine the cumulative effect of thousands of variants with small individual effects that are not significant when considered individually. These measurements, known as polygenic scores (PGS), have emerged as a valuable tool for identifying groups of patients at extreme risk or protected from developing the disease. These scores can be as high as the risk attributable to monogenic variation.^11^ PGS have been studied in PD^12^ as well as in common PD comorbidities, such as diabetes,^13^ depression^14^ and epilepsy,^15^ among others.^16,17^ However, while genetic risk factors for PD have been extensively investigated, the genetic basis of comorbidities within PD remains largely unexplored.

We hypothesized that genetic predisposition to comorbidities, as measured by PGSs, differs between PD patients and the general population, potentially due to PD-related environmental stressors amplifying their manifestation. While our findings indicate that comorbidity-PGSs do not significantly differ between PD patients and the general population, we show that comorbidity-PGSs are associated with age at onset (AAO) and sex-specific disease dynamics in PD patients. Our findings highlight the importance of comorbidity genetics not only in the general population but also in shaping clinical heterogeneity within PD.

## Materials and methods

### Study design and analytical plan

We conducted our study in three steps: (1) built PGS for Type 2 diabetes, depression, migraine and epilepsy, selecting the optimum *P*-value threshold in an 80% train/20% validate split; (2) tested each PGS for association with: (a) PD cases versus general population controls; (b) PD cases with versus without the comorbidity; and (c) the highest 20% versus lower 80% of the PGS distribution, using the covariate-adjusted logistic regression; (3) repeat step 2 after stratifying PD cases by age at onset (<50, 50–70, > 70 years) and by sex, and displayed temporal patterns with cumulative-incidence curves.

### The UK Biobank and cohort configuration

The UK Biobank is a prospective cohort from the UK, which began recruiting participants in 2006.^18^ As of April 2024, the dataset included a total of 502 187 individuals. Participants’ ages within this cohort range from 40 to 69, and the study encompasses a follow-up period of up to 15 years. The dataset offers a diverse array of phenotypic and health-related information for each participant, encompassing lifestyle data, biological measurements, biomarkers and comprehensive body and brain imaging. We identified individual disease diagnosis using available International Classification of Diseases 10th Revision (ICD10) Codes.^19^ Namely, PD (G20), Type 2 diabetes (E11), major depressive disorder (MDD) (F32), migraine headaches (MH) (G43) and epilepsy (G40). For each comorbidity-specific analysis, we compared PD patients with the given comorbidity to those without it. The latter group may include individuals who have one or more of the other comorbidities but do not have the specific comorbidity under evaluation. We performed a comparison with the general population, generating the respective case/control configuration.

### Cohort quality control

Imputed genotypic data were retrieved from the UK Biobank data field 21 007. Quality control (QC) and imputation of single nucleotide variants (SNVs) were performed by Bycroft *et al.*^18^ and NHLBI Trans-Omics for Precision Medicine Consortium *et al*.^20^, respectively. SNV–QC—included variants with a call rate >95%, minor allele frequency (MAF) > 0.01, and variants with a deviation from the Hardy–Weinberg equilibrium with a *P* < 0.001. We selected imputed SNVs that had *R*^2^ > 0.3. We removed heterozygosity outliers and samples with discordant sex status from the imputed data. Then, we identified related individuals with a kinship coefficient (data field 22 021) greater than 0.0442 and removed one of them. Finally, we removed ancestry outliers based on the principal component analysis (data field 22 009) to ensure that only individuals of European ancestry were included in our analysis. This decision was made to reduce population stratification biases, as the GWAS summary statistics (SS) used for the PGS calculations were derived predominantly from European ancestry cohorts. Additionally, the limited sample sizes of non-European populations in the UK Biobank restricted statistical power for ancestry-stratified analyses.

### Genome-wide association studies summary statistics curation

We obtained GWAS summary statistics for T2D, MDD, MH and EPI from Mahajan *et al*.,^21^ Howard *et al*.,^22^ Gormley *et al*.^23^ and Stevelink *et al*.,^24^ respectively. We used summary statistics for ‘number of children ever born’ (NEB) from Barban *et al*.^25^ to generate a negative control polygenic score (NEB-PGS) to assess whether the observed associations were specific to the comorbidity-PGSs or if any PGS could be associated with comorbidity presentation in PD patients. As a reproductive behavior trait, NEB is not biologically related to the comorbidities assessed in this study. To address potential bias arising from overlapping data between individuals in the UK Biobank and those who participated in the respective GWASs, we implemented distinct strategies to remove the UK Biobank individuals from GWAS summary statistics. In the case of MH, we exclusively relied on 23andMe, Inc. summary statistics to exclude individuals from the UK Biobank, mitigating the risk of overfitting. For MDD, we used meta-analysis results from Wray *et al*.^26^ and conducted a replication of the meta-analysis conducted by Howard *et al*.^22,^ excluding the UK Biobank cohort. Using these results in conjunction with 23andMe data, we performed the meta-analysis using METAL software^27^ to adjust the meta-analysis for the effective sample size. For T2D, since the summary statistics provided by Mahajan *et al*.^21^ encompassed the UK Biobank data, we employed the R package Metasubstract^28^ to simulate a meta-analysis that excluded the UK Biobank individuals. For epilepsy, no corrections are necessary as the data did not include the UK Biobank individuals.

### PGS calculation

We generated PGSs based on the overlap between the UK biobank SNVs-QC and those reported in the SS of each comorbidity GWAS, namely, T2D,^21^ MDD,^22^ MH^23^ and epilepsy^24^ (Supplementary Table 1). We calculated comorbidity-PGSs as previously described, with minor modifications.^29^ Briefly, we eliminated duplicated and ambiguous SNVs (A/T or C/G) and pruned the UK Biobank data into a subset of independent SNVs (*r*^2^ < 0.1 within 500 kb of the most significant SNVs in GWAS SS). We generated PGSs for each individual using the allelic score function available in Plink 1.9 software^30^ using SNVs weights from the GWAS summary statistics previously indicated.^21-24,31^ We calculated PGS at different thresholds of *P*-values. We used PGS as a prediction tool for comorbidities and chose *P*-value thresholds that maximize the explained variance of the model in a validation data set (split cohort in 80% training and 20% validation). We calculated a PD-PGS using summary statistics from Nalls *et al*.,^10^ which included data from 23andMe. The methodology for generating the PD-PGS followed the same approach as for the comorbidity-PGSs, using pruning and thresholding based on GWAS significance levels. We evaluated the risk of comorbidities using two strategies: first, we conducted logistic regression analyses to assess the relationship between the comorbidity status and PGS deciles in PD patients. The analysis was adjusted for age, sex, four principal components (PC) 1–4 and Townsend deprivation index (TDI), with the first decile as the reference point.^29^ Second, we compared the top distribution (20%, 10%, 5%, 1%) with the rest of the population using the same logistic regression as mentioned above.^11^

### PGS standardization

To calculate the empiric PGS *P*-value threshold that explains more variability, we performed a logistic regression of comorbidity status against PGS adjusted for sex, age, four PC and TDI.^32^ To assess the explained variance, we calculated Nagelkerke’s pseudo-*R*^2^ in each *P*-value threshold by comparing the full model with the null model (without PGS).^29^ To compare different PGSs, we performed a normalization of the PGS obtained. We calculated the mean and standard deviation (SD) for the controls in all cases and used them to normalize the entire cohort. We normalized the data, assuming a normal distribution by subtracting the mean and dividing by the SD.^29^ We used PGS to compare the distribution between cases and controls of comorbidities in the general population and within the PD subset. For correlation analysis, all PGS were treated as a continuous variable. For subgroup comparisons, we stratified the PGS into deciles, following standard PGS reporting practices.^11^ To assess whether comorbidity-related genetic risk influences PD presentation, we stratified cases by AAO (<50, 50–70, > 70 years) and compared PGS distributions across groups. This stratification was based on the prior evidence suggesting that the early-onset (<50, EOPD) and late-onset (>50, LOPD) PD may have distinct genetic and clinical characteristics. These comparisons were also stratified by sex. Logistic regression models were used to compare odds ratios (OR) across groups. We assessed the prevalence of comorbidities and PD based on the onset of PD and comorbidities, respectively. Furthermore, we compared the prevalences between the low PGS group (deciles 1 and 2) and the high PGS group (deciles 9 and 10).

### Statistical analyses

We conducted a t-test to assess differences between the comorbidity cases and controls in the PD subset and calculated OR using the logistic regression, adjusting for age, sex, the first four PC and TDI, with 95% confidence intervals (CIs). To correct for multiple testing, we applied the Bonferroni correction. Additionally, we conducted a *post hoc* power calculation to evaluate the statistical power of our analyses, particularly for subgroup comparisons. Given the large sample size of the general population (*n* = 370 480) and PD cases (*n* = 4144), we had sufficient power (>99.9%) to detect moderate effect sizes (odds ratio ≥1.3) when comparing PD cases with comorbidities and without. For the AAO comparisons (50–70 versus > 70 years, *n* = 1650 versus *n* = 2352), power remained high at 99.5%. The sex-stratified analysis (60% male, 40% female in PD cases) had lower but adequate power (88.7%) to detect sex-related differences with odds ratios ≥1.2. These calculations indicate that our study was well-powered to detect moderate to strong associations, though smaller effects in subgroup analyses may require larger sample sizes for confirmation.

All statistical analyses were conducted in R version 4.2.2,^33^ and visualizations were generated using the ggplot2 R library.^34^

## Results

**Figure 1:**
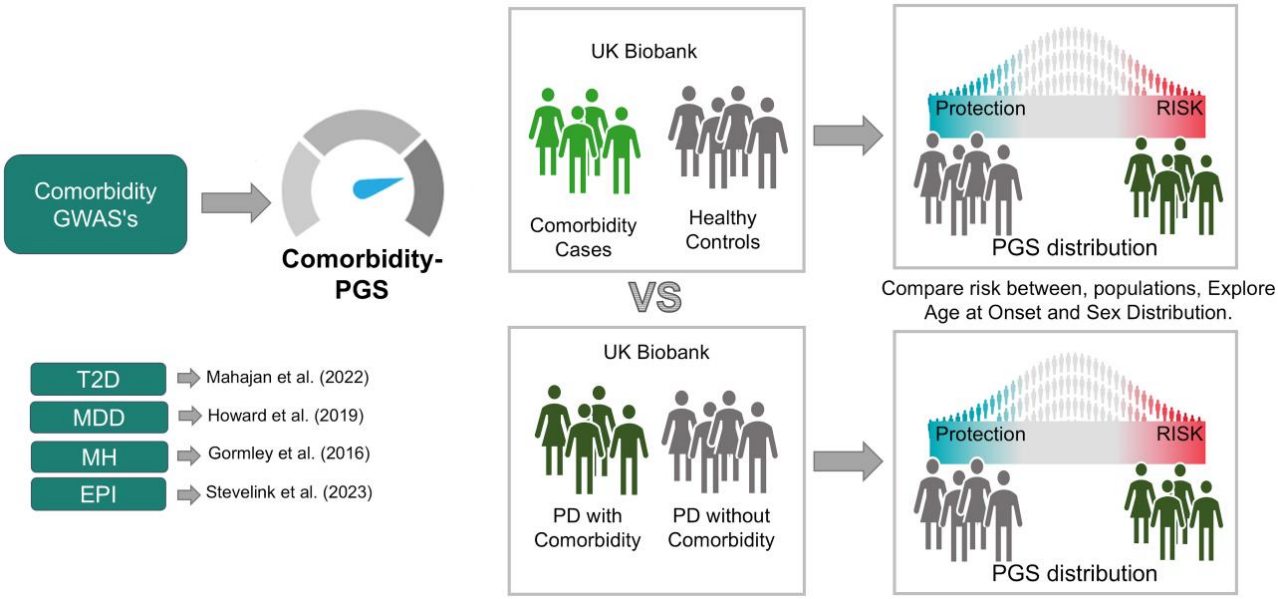
**Study overview:** schematic approach to assess the role of PGS associated with various comorbidities in shaping its manifestation within the context of PD. The methodology involves the analysis of type 2 diabetes, major depressive disorder, migraine headaches and epilepsy. The study explores the complex interaction between the genetic factors and the appearance of these comorbidities. By examining comorbidity-PGS profiles at different stages of PD onset and considering gender-specific patterns, the methodology aims to uncover insights into the nuanced genetic architecture that underlies the coexistence of PD and specific comorbidities.

We studied how common genetic variants associated to different comorbidities affect the risk of PD patients developing those comorbidities. We used available GWAS summary statistics to calculate PGSs for the selected comorbidities in PD patients from the UK Biobank. Our approach is illustrated in Fig. 1. We focused on four comorbidities: T2D,^21^ MDD,^22^ MH^23^ and EPI^24^ (Supplementary Table 1). After QC checks (see Methods), the final UK Biobank cohort consisted of 374 624 individuals, including 4144 PD cases and 370 480 general population participants. Demographic characteristics and comorbidity distributions are described in Table 1.

**Table 1:** Cohort description

Diagnosis (ICD10)	Group	*n*	Age	Male (%)	*n*	Age	Males (%)
		General population			Parkinson’s disease		
Type 2 diabetes (F32)	Controls	344 423	57 ± 8	154 329 (44.8)	3493	63 ± 5	2139 (61.2)
	Cases	29 841	61 ± 7	17 955 (60.2)	651	63 ± 5	455 (69.9)
Depression (E11)	Controls	351 576	57 ± 8	163 982 (46.6)	3379	63 ± 5	2173 (64.3)
	Cases	22 688	57 ± 8	8302 (36.6)	765	62 ± 5	421 (55.0)
	Cases						
Migraine headache (G43)	Controls	368 530	57 ± 8	170 825 (46.3)	4061	63 ± 5	2564 (63.1)
	Cases	5734	56 ± 8	1459 (25.4)	83	61 ± 7	30 (36.1)
Epilepsy (G40)	Controls	368 131	57 ± 8	169 145 (45.9)	3945	63 ± 5	2465 (62.5)
	Cases	6133	57 ± 8	3139 (51.2)	199	62 ± 6	129 (64.8)
All cohort	All	374 624	57 ± 8	172 284 (46.0)	4144	63 ± 5	1757 (62.0)

### High comorbidity-PGS are associated with comorbidity presentation in PD patients

**Figure 2:**
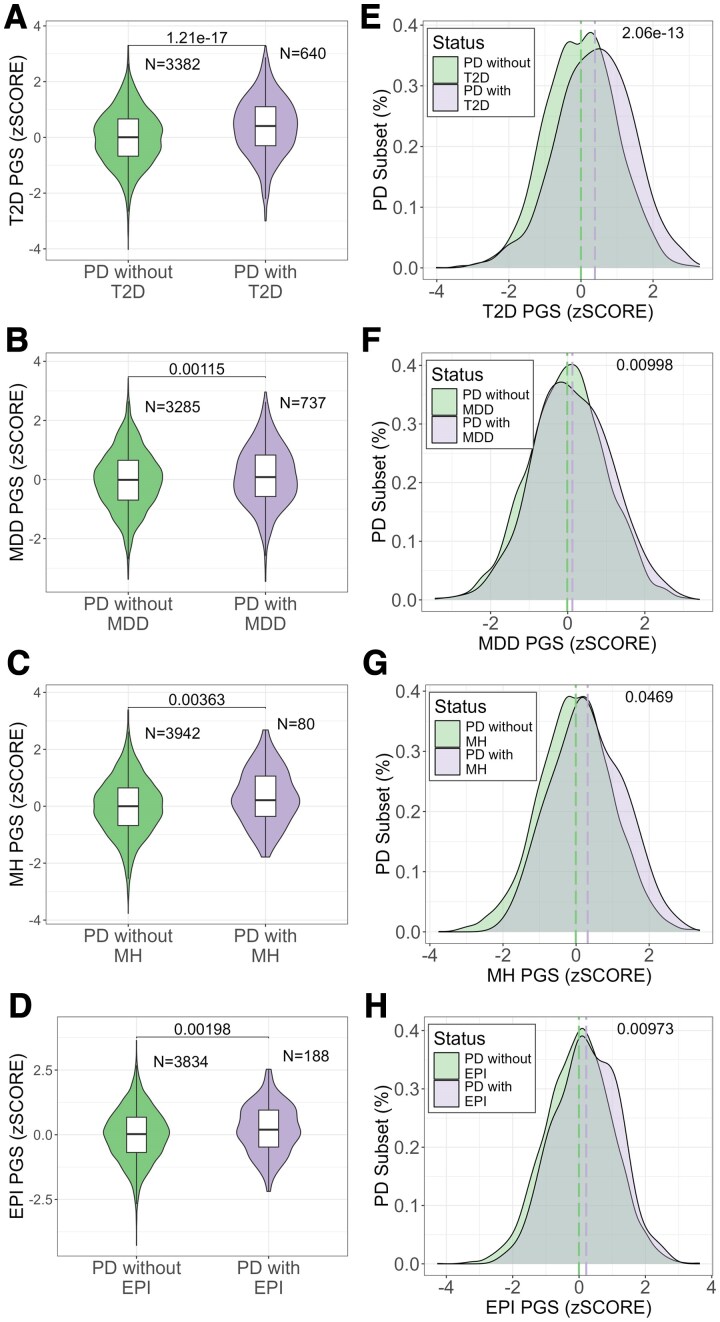
**Comorbidity-PGS in PD patients from the UK Biobank:** (**A–D**) PGS comparison between PD and PD with (**A**) type 2 diabetes (*n* = 640, *T* = −8.74, *P*-value = 1.21 × 10^−17^), (**B**) major depressive disorder (*n* = 737, *T* = −3.26, *P*-value = 0.0115), (**C**) migraine headache (*n* = 80, *T* = −2.99, *P*-value = 0.00363), (**D**) epilepsy (*n* = 188, *T* = −3.13, *P*-value = 0.00198). *P*-values were obtained with the t-student test for group comparison. Each group shows the number of individuals included in each group. (**E–H**) PGS distribution between the PD only subset and PD with (**E**) type 2 diabetes (*n* = 640, *D* = 0.166, *P*-value = 2.06 × 10^−13^), (**F**) major depressive disorder (*n* = 640, *D* = 0.0663, *P*-value = 0.00998), (**G**) migraine headache (n = 640, *D* = 0.154, *P*-value = 0.0469), (**H**) epilepsy (*n* = 640, *D* = 0.122, *P*-value = 0.00973). The dashed lines indicate the mean for each group. The *P*-value was calculated with the Kolmogorov–Smirnov to compare the normal distribution between the groups.

We identified significant differences in the mean comorbidity-PGS relationship between the PD cases with and without each comorbidity, namely, T2D (*P*-value: 1.21 × 10^−17^), MDD (*P*-value: 1.15 ×10^−3^), MH (*P*-value: 3.63 ×10^−3^) and EPI (*P*-value: 1.98 ×10^−3^) (Fig. 2A–D). Overall, PD cases with T2D (*P*-value: 2.06 ×10^−13^), MDD (*P*-value: 9.98 ×10^−3^), MH (*P*-value: 4.69 ×10^−2^) and EPI (*P*-value: 9.73 × 10^−3^) exhibited higher PGSs compared to PD cases without these comorbidities (Fig. 2E–H). As expected, the NEB-PGS showed no association with any of the comorbidities studied (Supplementary Fig. 1), supporting the specificity of the primary PGS associations.

To explore the relationship between the comorbidity-PGS and the likelihood of developing comorbidities in PD patients, we created decile plots. While a general trend is observed, fluctuations in the decile-based ORs, particularly for EPI and to a lesser extent for MDD and MH, indicate that the relationship is not strictly linear (Supplementary Fig. 2).

We calculated the OR for individuals in the top 20%, 10%, 5% and 1% of the PGS distributions (Table 2). For T2D, we observed a consistent increase in risk across all thresholds (OR: 20%: 2.19; 10%: 2.40; 5%: 2.62; 1%: 3.51). For MH and MDD, statistical significance was observed at the 20% and 5% thresholds, but not at the 10% or 1% levels. (MH OR: 20%: 1.76–10%: 1.79–5%: 2.51–1%: 2.33; MDD OR: 20%: 1.34–10%: 1.27–5%: 1.52–1%: 1.34). For EPI, only the 20% threshold was significant (OR = 1.58, *P* = 8.45 × 10⁻³). Based on these findings, we used the 20% threshold consistently in all subsequent analyses. Next, we compared the comorbidity-PGS risk of PD individuals with the general population. This analysis was conducted separately within each subset, where the OR was calculated by comparing individuals in the top 20% PGS group against the remaining 80%. We did not observe significant differences in comorbidity-PGS between PD patients and the general population for any of the investigated comorbidities (Supplementary Fig. 3).

**Table 2:** Comorbidity-PGS for the extreme distribution in individuals with PD from the UK Biobank

	OR	95% CI	*P*-value	Case/Control high PGS	Case/Control low PGS
**T2D-PGS**					
20% top PGS	2.19	1.81–2.65	1.27 × 10^−15^	200/585	432/2706
10% top PGS	2.40	1.89–3.05	6.68 × 10^−13^	114/279	518/3012
5% top PGS	2.62	1.91–3.59	2.46 × 10^−09^	62/135	570/3156
1% top PGS	3.51	1.85–6.67	1.29 × 10^−04^	16/24	616/3267
**MDD-PGS**					
20% top PGS	1.34	1.11–1.63	3.01 × 10^−03^	171/612	543/2597
10% top PGS	1.27	0.982–1.65	0.0687	84/309	630/2900
5% top PGS	1.52	1.08–2.13	0.016	48/149	666/3060
1% top PGS	1.34	0.631–2.84	0.448	9/31	705/3178
**MH-PGS**					
20% top PGS	1.76	1.07–2.89	0.0251	24/761	52/3086
10% top PGS	1.79	0.967–3.3	0.0639	13/380	63/3467
5% top PGS	2.51	1.22–5.14	0.0123	9/188	67/3659
1% top PGS	2.33	0.544–9.95	0.255	2/38	74/3809
**EPI-PGS**					
20% top PGS	1.58	1.12–2.22	8.45 × 10^−03^	52/733	131/3007
10% top PGS	1.11	0.691–1.78	0.666	21/372	162/3368
5% top PGS	1.04	0.539–2.02	0.902	10/187	173/3553
1% top PGS	1.59	0.482–5.22	0.448	3/37	180/3703

Finally, we assessed the correlation between comorbidity-PGS scores for different comorbidities, and found no significant correlations among PGS values, suggesting independent genetic contributions to the manifestation of distinct comorbidities in the population with PD (Supplementary Fig. 4). We also checked whether the PD-PGS correlated with any comorbidity-PGS. We observed that the PD-PGS does not correlate with the PGS of the different comorbidities (Supplementary Fig. 5).

### Heterogeneous associations of comorbidity-PGSs with PD Onset

**Figure 3:**
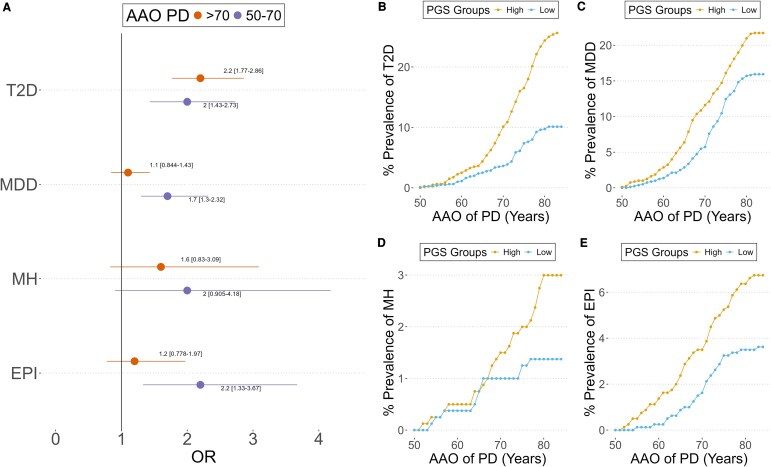
**Association of comorbidity-PGSs with the PD onset in individuals from the UK Biobank:** (**A**) shows are the OR and the 95% confidence interval for PD comorbidities in the PD subset, 50–70 (lower line/dot) and >70 (upper line/dot) age at onset. The OR was obtained with a logistic regression for comorbidity using age, sex and the first four PC as covariates comparing 20% of high PGS with the rest of the group in each age interval. A total of 2352 PD cases with AAO > 70 and 1571 with AAO between 50 and 70 was included (T2D > 70: 417 cases, 50–70: 215; MDD > 70: 408, 50–70: 306; MH > 70: 45, 50–70: 31; EPI > 70: 108, 50–70: 75). (**B–E**) Prevalence of (**B**) type 2 diabetes, (**C**) major depressive disorder, (**D**) migraine headache, (**E**) epilepsy as a function of the onset of PD for PGS strata. Each point represents the percentage of individuals with the comorbidity at a given age of the PD onset. Curves are shown separately for the low PGS group (0–20%, blue, *n* = 785) and the high PGS group (80–100%, orange, *n* = 785). No statistical test was applied; this is a descriptive analysis.

To assess whether comorbidity-PGS contributes to the onset of PD, we conducted a linear regression analysis of PGS and AAO. The results revealed no correlation between the comorbidity-PGS and the AAO of PD (Supplementary Fig. 6). We analysed the top 20% distribution within different AAO ranges (Supplementary Table 2). When assessing the OR in the group with onset before the age of 50 (*n* = 142), no significant results were found for any comorbidities (Supplementary Table 2). Next, we analysed individuals who developed symptoms after the age of 50 (*n* = 4002) and divided them into two groups: ages 50–70 (*n* = 1650) and ages over 70 (*n* = 2352). We found differences in the risk of T2D in both age groups (50–70: OR: 1.98, *P*-value: 3.9 × 10^–05^; > 70: OR: 2.25, *P*-value: 3.43 × 10^–11^). This suggests that the genetic risk score for T2D may not be differentially associated with the onset of PD. However, we observed an increased risk of MDD (50–70: OR: 1.74, *P*-value: 2.22 × 10^−04^; >70: OR:1.10, *P*-value: 0.483) and EPI (50–70: OR: 2.21, *P*-value: 2.32 × 10^−03^; >70: OR: 1.24, *P*-value: 0.366) only in the 50–70 age group, indicating a higher genetic risk for MDD and EPI in individuals with LOPD who develop symptoms at an earlier stage within this category. We did not find a significant association for MH between the age groups (50–70: OR: 1.95, *P*-value: 0.0883; >70: OR: 1.60, *P*-value: 0.160) (Supplementary Table 2; Fig. 3A). To account for age-related effects independent of PD onset, we conducted the same analysis in the general population, using age at sampling as the stratification variable. This analysis (Supplementary Table 3, Supplementary Fig. 7) showed that odds ratios for comorbidity-PGS remained stable between individuals younger than 50 and those aged 50–70 and are not significant in >70 years old, indicating that genetic risk for these comorbidities does not vary significantly with age in the general population. In individuals over 70, CIs were wider due to low sample size, limiting the power to detect associations in this group. These findings suggest that the observed differences in PD are not simply a reflection of general population trends but are specific to PD onset dynamics.

Next, we examined the temporal interaction between the age at PD onset and comorbidity incidence in different comorbidity-PGS strata. We note that most PD cases developed their comorbidities before the onset of PD: 67.3% of PD cases developed T2D before PD, 54.2% for MDD, 80.4% for MH and 59.3% for EPI. Subsequently, we evaluated how the accumulated prevalence of comorbidities changed in relation to PD onset in groups with high and low PGS. In the high PGS group, the prevalence of T2D shows a gradual increase around the ages of 55 and 65 years, rather than distinct peaks. For MDD, the prevalence in the low PGS group remains relatively stable until around 75 years of age. The prevalence of MH follows a similar trajectory in both groups until approximately age 65, after which the rate of increase slows down in the low PGS group, whereas it continues rising in the high PGS group. Additionally, for MH, the high PGS group shows a steeper increase starting at the age of 50, which becomes more pronounced after 70 years (Fig. 3).

Looking at the accumulated prevalence at the final stage. We observed that in the high PGS group, the prevalence of T2D was 25%, significantly higher than the 10% observed in the low PGS group. Similarly, the prevalence of MDD was 22% in the high PGS group, compared to 16% in the low PGS group. For MH, the prevalence was 3% in the high PGS group, compared to 1.4% in the low PGS group. Lastly, the prevalence of EPI was 4.9% in the high PGS group, compared to 2.8% in the low PGS group (Fig. 3).

### Comorbidity-PGS associations with sex-specific patterns

**Figure 4:**
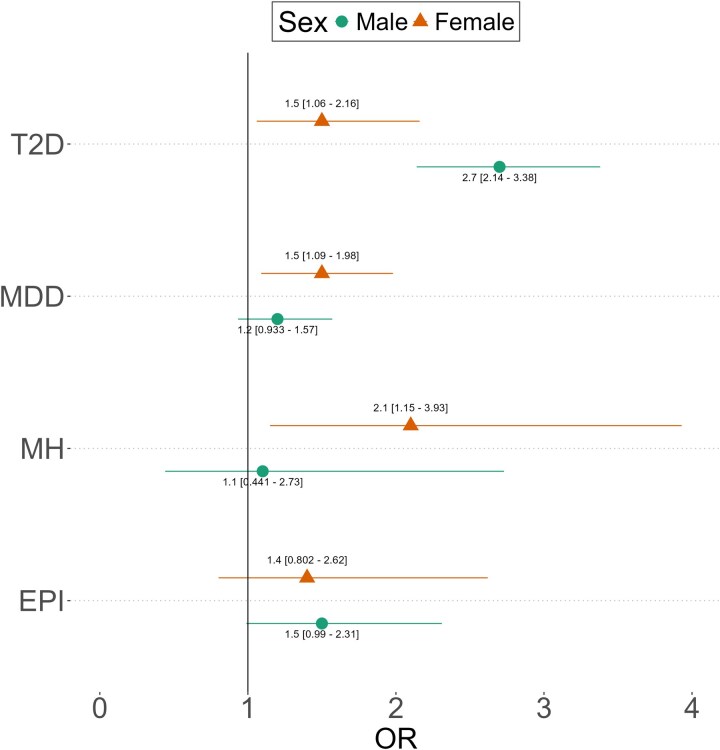
**Association of comorbidity-PGS with sex patterns.** Shows are OR and 95% confidence interval, for PD comorbidities in PD patients analysed by sex (males as circles and females as triangles). OR was obtained using the logistic regression for comorbidities with age, sex and the first four PC as covariates compared to 20% of the high PGS groups. A total of 2466 males and 1457 females with PD were included (T2D: males 446 cases, females 186; MDD: males 401, females 292; MH: males 28, females 48; EPI: males 120, females 63).

In the PD population, we found distinct sex-related dynamics. For MDD, the odds ratio for female patients was 1.47 (*P*-value: 0.0119), while male patients showed no association (OR: 1.21, *P*-value: 0.151). Similarly, for MH, the odds ratio for female patients was 2.13 (*P*-value 0.0155), while male patients showed no association (OR: 1.10, *P*-value: 0.843). In contrast, for T2D, both female and male patients are associated with an increased risk, but male patients have a higher risk compared to female patients (female: OR: 1.51, *P*-value: 0.0239; male: OR: 2.69, *P*-value: 2.11 ×10–17). No difference was observed for EPI (female: OR: 1.45, *P*-value: 0.219; male: OR: 1.51, *P*-value: 0.0554) (Supplementary Table 4, Fig. 4).

## Discussion

Our study shows that individuals with PD and concurrent comorbidities displayed elevated PGS values when compared to those without such comorbidities (Fig. 2), highlighting the role of common genetic susceptibility in the manifestation of these health challenges.^35,36^

Our study initially hypothesized that genetic predisposition to comorbidities, as measured by PGS, would be higher in individuals with PD compared to the general population. This assumption was based on the idea that PD-related physiological stressors or underlying disease mechanisms could amplify the manifestation of comorbidities in genetically predisposed individuals. However, our results indicate that comorbidity-PGSs do not significantly differ between PD patients and the general population, suggesting that these conditions arise independently of PD status rather than being exacerbated by the disease itself. This finding challenges the notion that genetic risk for comorbidities interacts with PD-specific pathophysiology to increase comorbidity burden beyond what is expected based on genetic predisposition alone.

Furthermore, we found that comorbidity-PGSs were also not correlated with PD-PGS, further reinforcing the independence of genetic risk factors for PD and the investigated comorbidities. This suggests that while PD patients frequently exhibit these comorbidities, their occurrence is not driven by shared common genetic architecture with PD but rather by independent genetic risk factors. These findings align with prior evidence^32,37^ that many comorbidities observed in PD patients are also highly prevalent in the general population, implying that their co-occurrence may be due to overlapping epidemiological and environmental factors rather than a direct genetic link to PD itself. It is important to note that individuals in the comparator groups may still present other comorbidities. However, since each polygenic score was constructed for a specific trait and the correlation between comorbidity-specific PGSs was low, we do not expect major interaction effects that would substantially bias the comparisons. Additionally, restricting the control group to individuals without any of the studied comorbidities would have considerably reduced the sample size, thereby limiting statistical power. The decile-based analysis of comorbidity-PGS in PD patients showed some fluctuations, particularly for EPI, suggesting that the relationship may not be strictly linear. This could be attributed to the lower number of PD cases with comorbidities, limiting statistical power for detecting strong correlations within the PD subset. Future studies with larger sample sizes, particularly for less prevalent comorbidities, such as EPI, will provide a more refined assessment of this relationship.

Given these findings, we shifted our focus towards understanding how genetic risk for comorbidities influences disease heterogeneity within PD. We observed that comorbidity-PGSs were associated with differences in disease onset and sex-specific risk patterns, suggesting that while genetic predisposition to comorbidities does not alter the likelihood of developing PD, it may influence how PD manifests in affected individuals. These results underscore the importance of considering individual genetic backgrounds when studying comorbidity patterns in PD, as the presence of certain genetic risk factors may help explain variation in disease onset and progression among patients.

We show a genetic susceptibility to T2D and EPI in individuals with LOPD who developed PD between 50 and 70 years old, suggesting that genetic predisposition may influence disease onset within this subgroup (Fig. 3). We also found sex-specific PGS patterns. While women with PD exhibited an elevated PGS for MDD and MH, male patients exhibited a greater risk for T2D (Fig. 4).

With the incorporation of clinical variables, comorbidity-PGS could be useful in delineating disease presentation in PD patients. For example, PGS for psychiatric comorbidities can be used as a predictor of these comorbidities in inflammatory bowel disease patients.^38^ In bipolar disorder patients, anxiety PGS influences anxiety comorbidity and suicidal behavior.^39^ In people with EPI-psychiatric conditions, PGS are associated with EPI presentation.^40^

AAO is a crucial factor in the risk of comorbidities associated with PD, influencing distinct patterns of comorbidity development. Our results show that the risk of EPI and MDD was concentrated in individuals with LOPD who developed symptoms between 50 and 70 years old. This suggests an elevated common genetic predisposition for these comorbidities in individuals who develop PD at an earlier stage within the LOPD category, rather than in EOPD.^6,41,42^ While recent studies highlight the association between T2D and PD presentation,^43-45^ our data indicate that this association does not contribute to a differential onset within LOPD, as no significant differences were observed between the 50–70 and >70 age groups.

Sex-specific associations also emerged as an important aspect of our findings. PD is 1.3 times more frequent in male than female patients.^46^ Furthermore, male patients are at greater risk for cognitive decline and motor symptoms, while female patients are at increased risk for non-motor symptoms.^47^ For MDD and MH, an increase in risk was observed among female patients (Fig. 4). For MH, no information on the PD risk between sexes was reported in the literature, but in the general population, the incidence of MH is higher in female patients.^48^ In PD, the incidence of MDD in female patients is greater than in male patients.^49^ This higher incidence may be explained, in some part, by the genetic contribution of PGS. On the contrary, our results show that T2D exhibited a higher risk in male patients compared to female patients. In this case, men with T2D are more prone to develop PD,^50^ suggesting that all this increased incidence in comorbidities may be explained, in some part, by the common genetic contribution.

Our work is in line with recent reports on PD comorbidities with large databases, such as the UK Biobank^32^ and 23andMe,^37^ which determined based on prevalence, the risk that PD patients have to develop comorbidities. However, our work goes further by exploring whether this risk is attributable to genetic burden as measured by the PGS. The PGS is an emerging methodology that can be used to define risk and, with more clinical validation or in combination with other biomarkers, may be useful and reliable predictor of disease in clinical practice.^51,52^

Although our study offers novel information, it is not without limitations. Quality and availability of genetic and phenotypic data. The UK Biobank data enable us to work with frequent comorbidities such as T2D (*n* = 640) and MDD (*n* = 737). In EPI and MH, the number of people with PD and these comorbidities is fewer (EPI = 180, MH = 80), limiting the power of the analytic results. Working with ICD10 codes to define phenotype limits the overall value of PGS for some diseases, including EPI.^53^ Furthermore, it is important to note that the UK Biobank data are predominantly derived from white British individuals, which limits the generalizability of the findings to other populations. Finally, we only evaluated common genetic variation, rare variations are also associated with the development of the evaluated comorbidities.^54-57^ Future studies evaluating common and rare variations will comprehensively assess the full genetic landscape of PD comorbidity presentation.^56,58^

PD is well-recognized for its clinical heterogeneity,^59^ making it challenging for individuals newly diagnosed to predict the specific subtype of PD they may develop.^60^ As a result, studies exploring comorbidities dynamics associated with PD, as this study, provide valuable insights into the diverse range of additional health issues that may co-occur with this condition.^61^ In conclusion, our results indicate that common genetic predisposition to type 2 diabetes, depression, migraine and epilepsy does not increase Parkinson’s disease risk. Instead, these polygenic burdens shift age at onset towards the 50–70 year window and produce distinct sex-linked patterns among affected individuals.

## Supplementary Material

fcaf325_Supplementary_Data

## Data Availability

The analysis code and scripts used in this study are openly available in the GitHub repository Laboratorio-de-Neurogenetica-Clinica/PGS-PD-Comorbidities.
