# Supplementary material for: Polygenic scores contribution to Parkinson’s disease comorbidities
Source: Brain Commun. 2025 Aug 29;7(5):fcaf325. doi: 10.1093/braincomms/fcaf325 (PMC12448616; doi:10.1093/braincomms/fcaf325)
Supplement: fcaf325_Supplementary_Data [file fcaf325_supplementary_data.pdf]

Supplementary figures

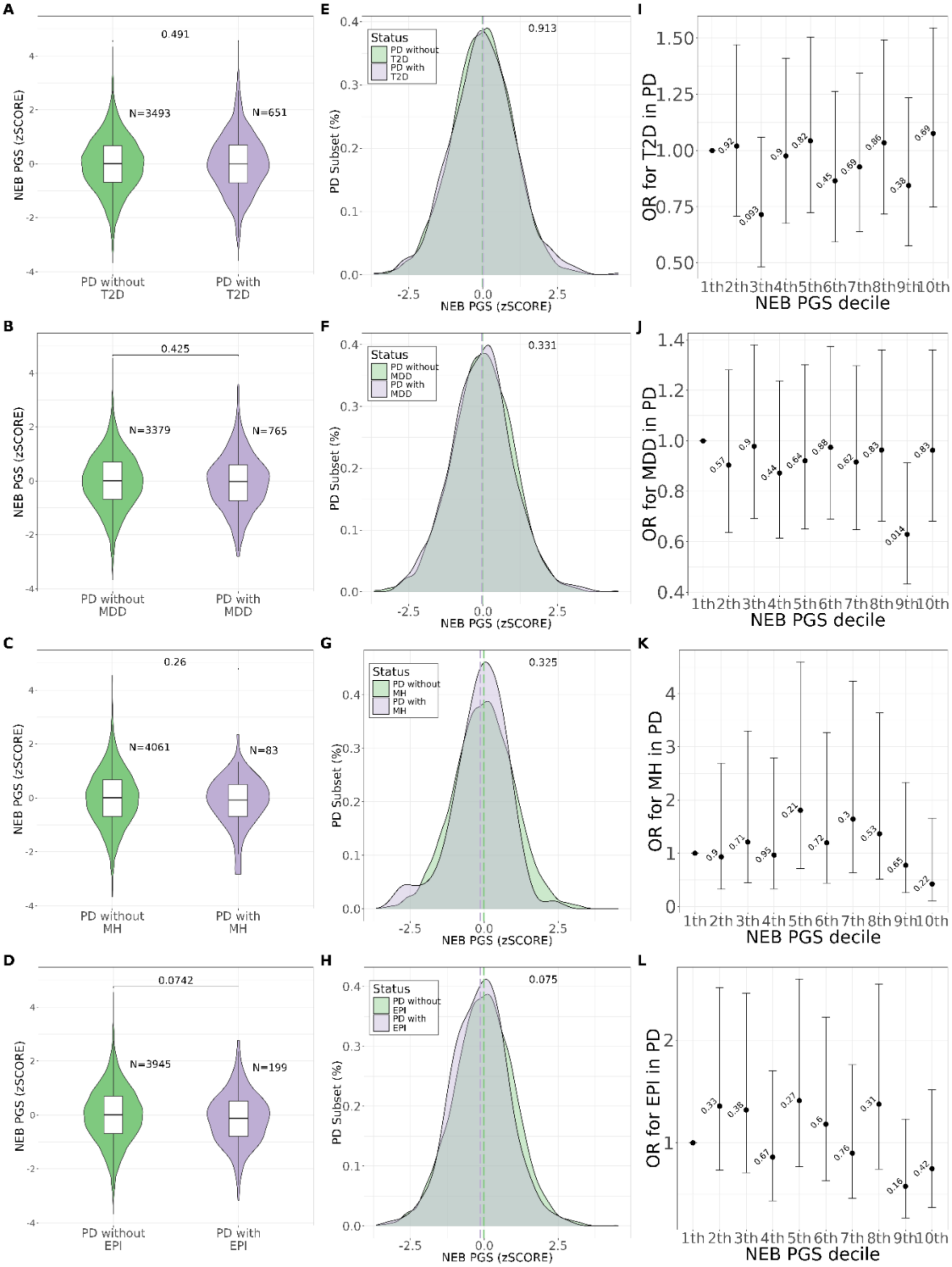

Supplementary Figure 1. Association of NEB-PGS with presentation in individuals from UK biobank: (A-D) PGS comparison between PD and PD with (A) type 2 diabetes (n= 640, T= 1.47, p-value= 0.141), (B) major depressive disorder (n= 737, T= -0.504, p-value= 0.614), (C) migraine headache (n= 80, T= 0.257, p-value= 0.798), (D) epilepsy (n= 188, T= 2.08, p-value= 0.0387). P-values were obtained with the t-student test for group comparison. Each group shows the number of individuals included in each group. (E-H) PGS distribution between the PD subset in green and PD with (E) type 2 diabetes (n= 640, D= 0.0431, p-value= 0.260), (F) major depressive disorder (n= 640, D= 0.0297, p-value= 0.639), (G) migraine headache (n= 640, D= 0.0786, p-value= 0.697), (H) epilepsy (n= 640, D= 0.0919, p-value= 0.0870) in purple. The colored lines indicate the mean for each group. The P-value was calculated with Kolmogorov-Smirnov to compare the normal distribution between the groups. (I-L) The odds ratio of the comorbidities of PGS deciles in PD. We assigned a subset of individuals based on the NEB-PGS score in 10 deciles with the same number of individuals. The odds ratio and the p-values were calculated with a logistic regression for comorbidities adjusted by age, sex, PC 1-4, and Townsend deprivation index. Decile 1 was used as a reference group. Each decile includes approximately 415 PD patients. The panels indicate comorbidities, (I) type 2 diabetes, (J) major depressive disorder, (K) migraine headache, (L) epilepsy. EPI: Epilepsy; MDD: Major depressive disorder; MH: Migraine headaches; OR: Odds Ratio; PD: Parkinson´s Disease; PGS: Polygenic Score; T2D: Type 2 diabetes

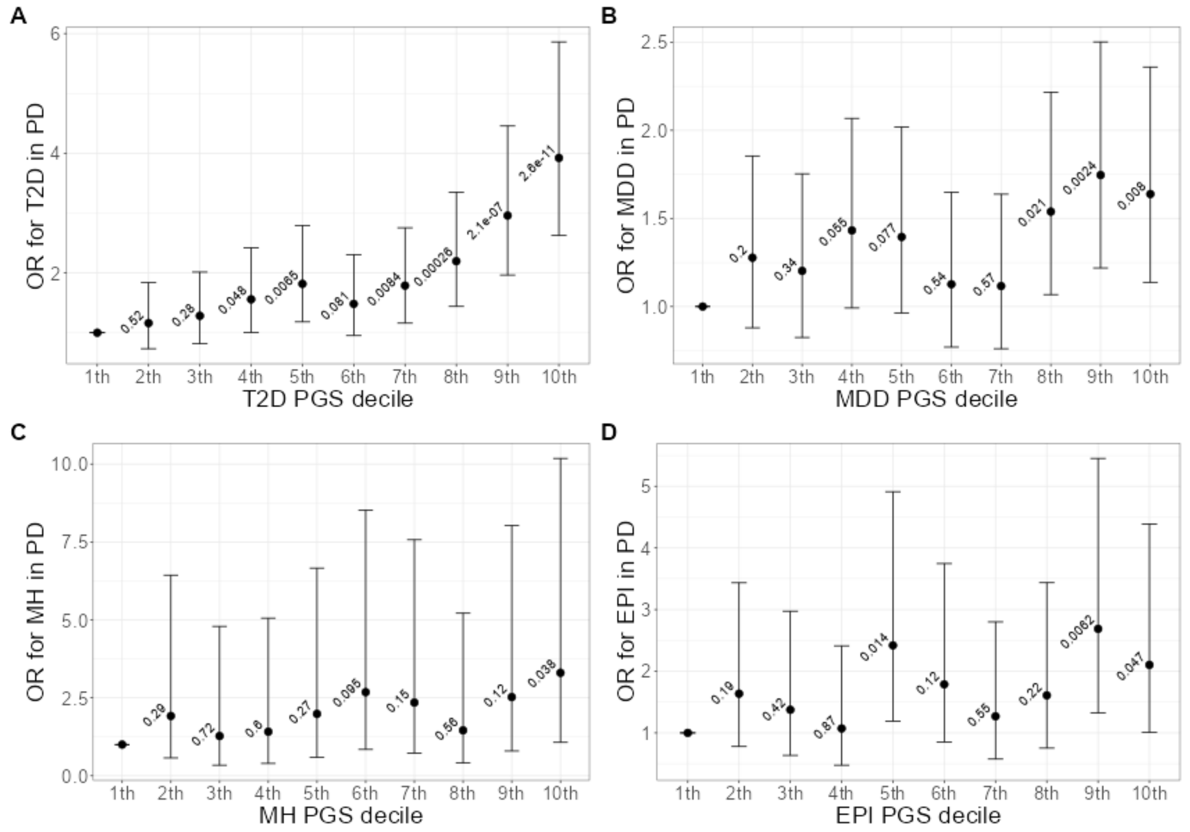

Supplementary Figure 2. Odds ratio of comorbidities by PGS deciles in PD individuals from UK Biobank: We assigned the PD subset of individuals based on the PGS score to 10 deciles with the same number of individuals. The odds ratio and the p-values were calculated with a logistic regression for comorbidities adjusted by age, sex, PC 1-4, and Townsend deprivation index. Decile one was used as a reference group. Each decile includes approximately 415 PD patients. The panels indicate comorbidities, (A) type 2 diabetes, (B) major depressive disorder, (C) migraine headache, (D) epilepsy. EPI: Epilepsy; MDD: Major depressive disorder; MH: Migraine headaches; OR: Odds Ratio; PD: Parkinson´s Disease; PGS: Polygenic Score; T2D: Type 2 diabetes

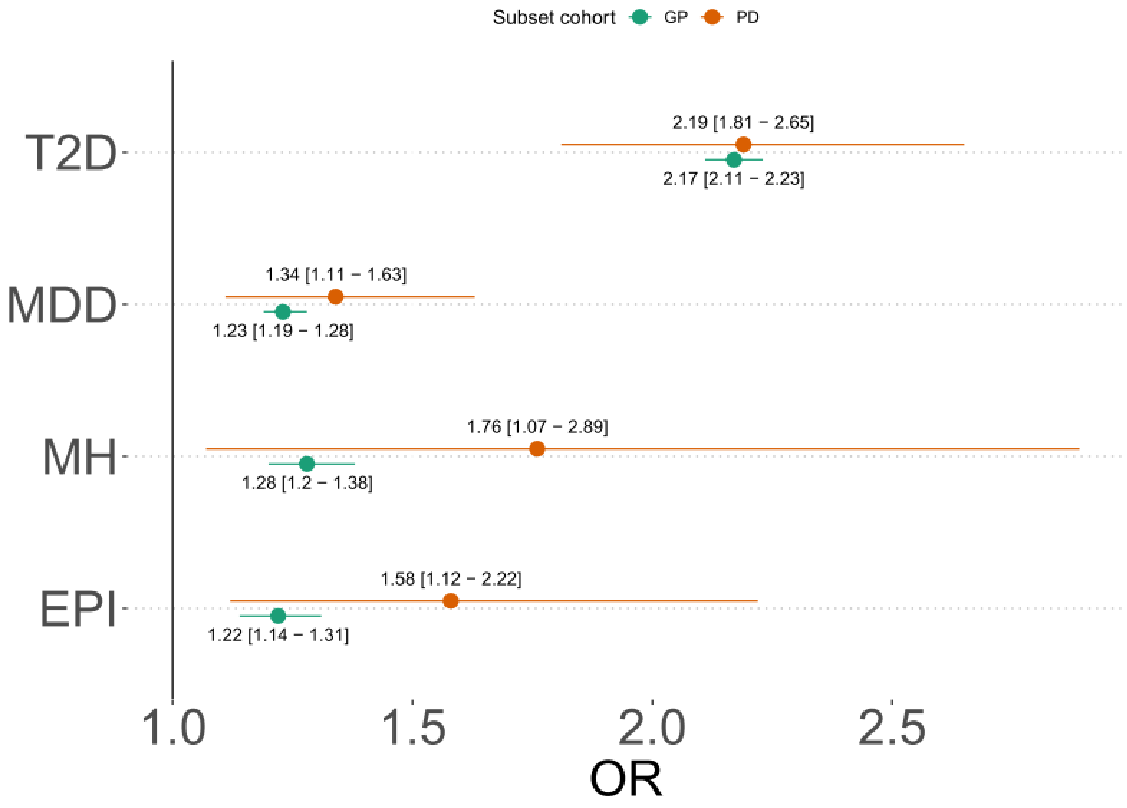

Supplementary Figure 3. Comorbidity-PGS in PD and General population from the UK Biobank: Shows the OR and the 95% confidence interval for PD comorbidities in the PD subset (orange) and the general population (green). OR was obtained using logistic regression for each comorbidity separately within the PD and general population subsets. The analysis compared individuals in the top 20% PGS group against the remaining 80%, adjusting for age, sex, and the first four principal components. In the PD subset, a total of 3,923 individuals were included (T2D: 632 cases; MDD: 714; MH: 76; EPI: 183). In the general population, 294,519 individuals were included (T2D: 26,610 cases; MDD: 17,387; MH: 4,289; EPI: 4,762) . EPI: Epilepsy; GP: General population; MDD: Major depressive disorder; MH: Migraine headaches; OR: Odds Ratio; PD: Parkinson´s Disease; T2D: Type 2 diabetes

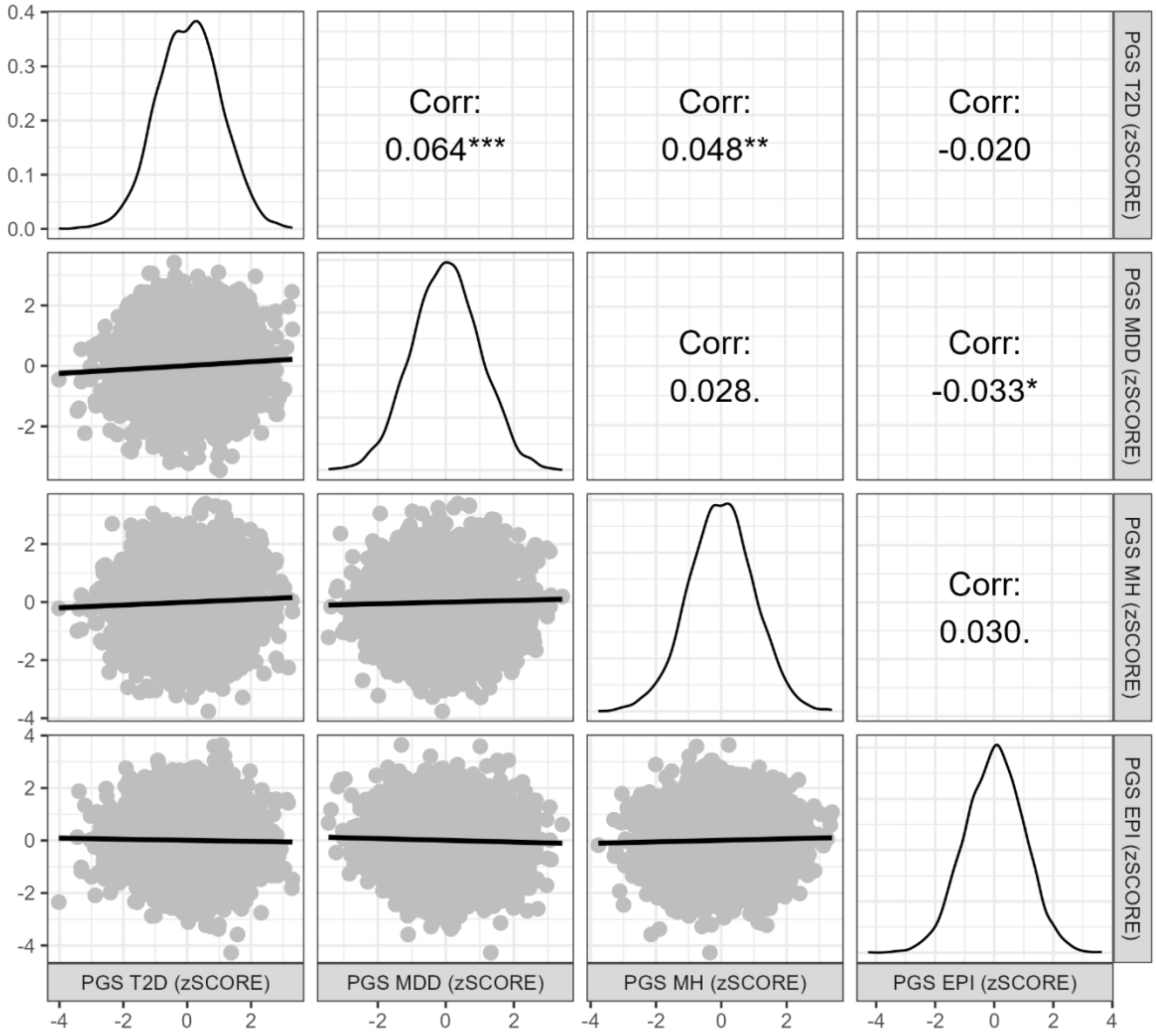

Supplementary Figure 4. Association between Comorbidity-PGS. The lower panel displays pairwise linear correlations between the polygenic scores (PGSs) of different comorbidities, with each point representing one individual. The PGS for one comorbidity is plotted against the PGS for another. Correlation analyses were performed within the PD group (n = 3,923). The diagonal shows the distribution of each individual PGS. The upper panel reports the Pearson correlation coefficient and the statistical significance of the linear regression between PGS pairs. Statistical significance levels: . ($p < 0.1$), * ($p < 0.05$), ** ($p < 0.01$), *** ($p < 0.001$), **** ($p < 0.0001$). EPI: Epilepsy; MDD: Major depressive disorder; MH: Migraine headaches; PGS: Polygenic Score; T2D: Type 2 diabetes.

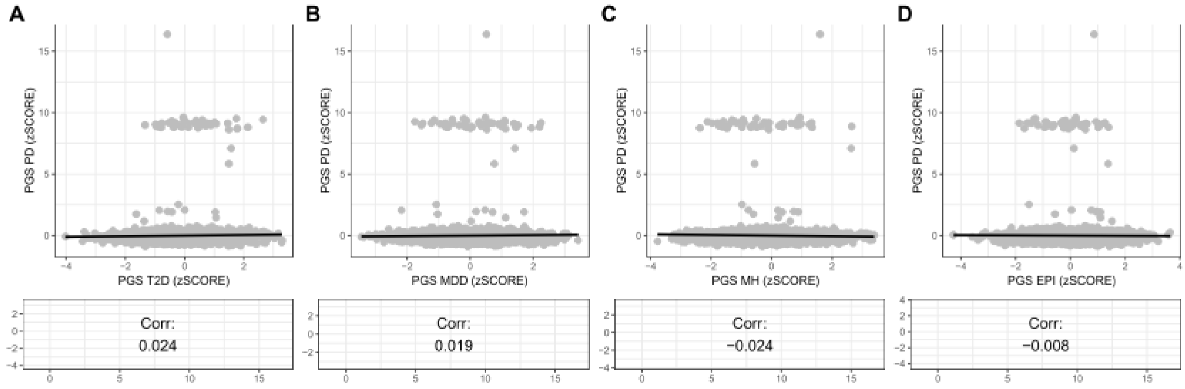

Supplementary Figure 5 Correlation between PD-PGS and comorbidity-PGS: The upper panel displays the linear correlations between PD-PGS and the PGSs of each comorbidity as (A) type 2 diabetes, (B) major depressive disorder, (C) migraine headache, (D) epilepsy. Each point represents one individual. Correlation analyses were performed within the PD group (n = 3,923). The lower panel reports the Pearson correlation coefficients. No correlations reached statistical significance. EPI: Epilepsy; MDD: Major depressive disorder; MH: Migraine headaches; PD: Parkinson's disease; PGS: Polygenic Score; T2D: Type 2 diabetes.

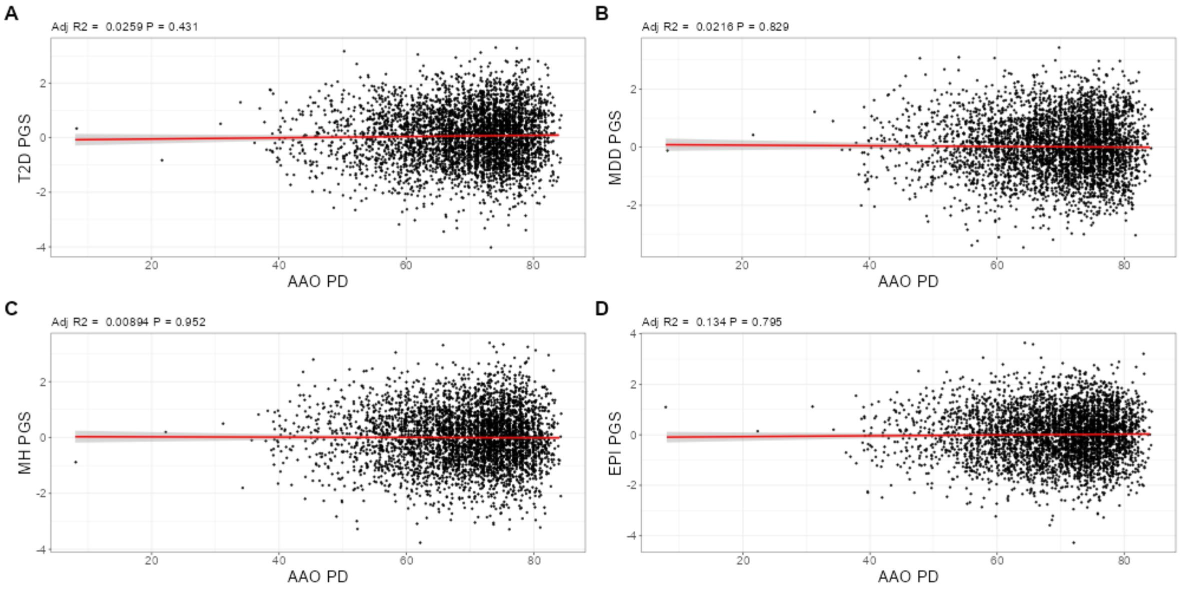

Supplementary Figure 6. Comorbidity-PGS correlation with age at onset of PD in PD cases of UK biobank: (A-D) Shows the relationship between comorbidity and age at onset of PD for (A) type 2 diabetes, (B) major depressive disorder, (C) migraine headache, (D) epilepsy. Each point represents one PD patient. The red lines indicate the model generated by the linear regression model adjusted by age, sex, PC 1-4, and Townsend's deprivation index. At the top of the plot, the Pearson correlation coefficient and the statistical significance of the linear regression are shown. Analyses were performed in the PD subset (n = 3,923). AAO: Age at onset; EPI: Epilepsy; MDD: Major depressive disorder; MH: Migraine headaches; PD: Parkinson´s Disease; PGS: Polygenic Score; T2D: Type 2 diabetes

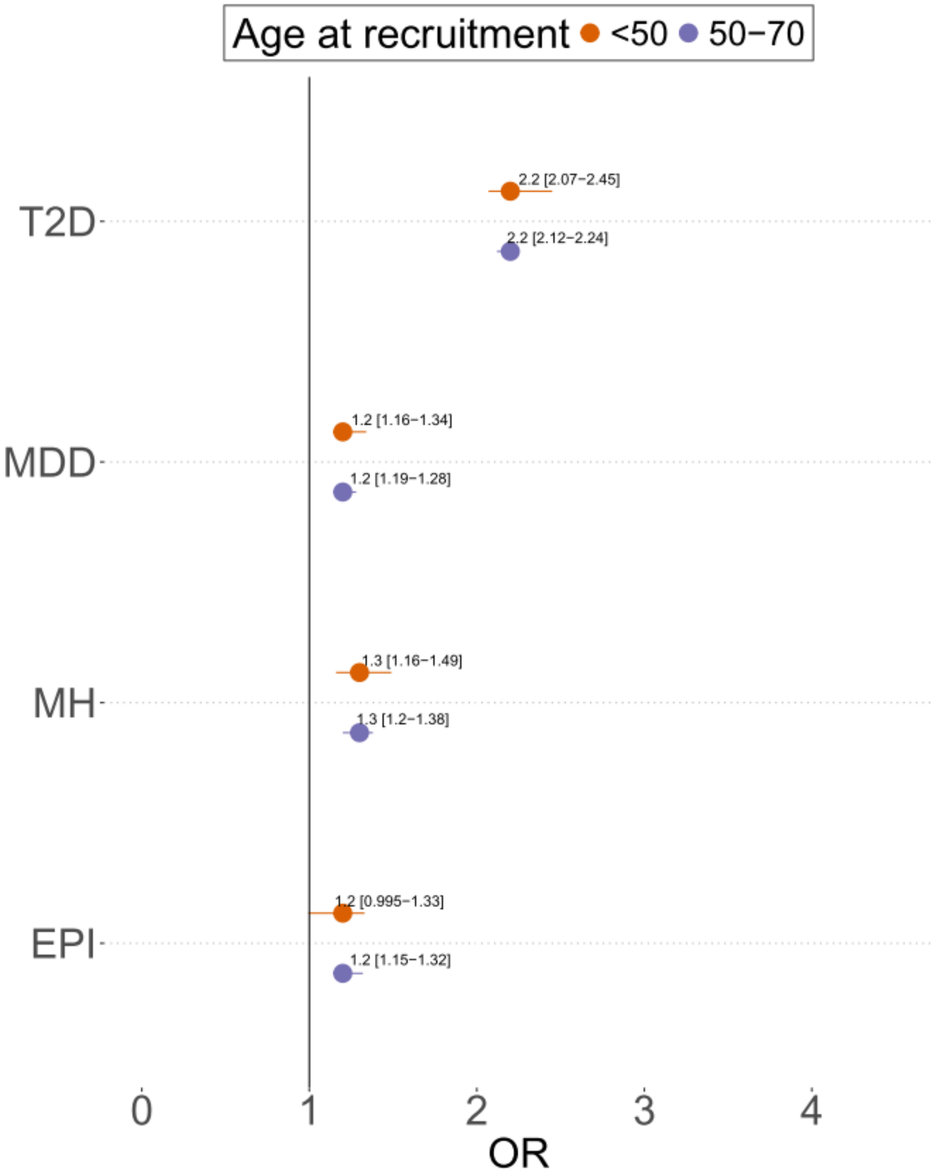

Supplementary Figure 7. Association of comorbidity-PGSs with age at recruitment in General population from the UK Biobank: Shows are the OR and the 95% confidence interval for PD comorbidities in the General population), <50 (Orange), and 50-70 (Purple) age at recruitment. OR was obtained with a logistic regression for comorbidity using age, sex and the first four PC as covariates comparing 20% of high PGS with the rest of the group in each age interval. A total of 75,720 individuals aged <50 and 296,422 aged 50–70 were included (T2D <50: 2,588 cases, 50–70: 26,971; MDD <50: 4,561; 50–70: 17,983; MH <50: 1,362, 50–70: 4,339; EPI <50: 1,180, 50–70: 4,904). EPI: Epilepsy; MDD: Major depressive disorder; MH: Migraine headaches; OR: Odds Ratio; T2D: Type 2 diabetes

Supplementary Tables

Supplementary Table 1. Source of Summary Statistics for Comorbidities.

Trait	Cases	Control	N SNVs	QC SNVs	Clumped SNVs	p-value Threshold	N SNVs PGS	Comments	Population	Ref.
**T2D**	180,834	1,159,055	10,454,875	8,896,334	357,713	0.05	40,926	Meta subtract UKB	Multi-Ancestry	[1]
**MDD**	246,363	561,190	11,965,230	10,258,741	347,847	0.05	43,913	Replicate metanalysis without UKB	European	[2]
**MH**	59,674	316,078	19,023,436	8,056,917	323,647	0.05	46,910	Only 23 and Me	European	[3]
**EPI**	29,944	52,538	4,904,664	4,1587,536	94,706	0.05	21,148	NA	Multi-Ancestry	[4]
**NEB**	343,072		2,470,444	2,054,459	136,422	0.05	17,988	NA	European	[5]

Supplementary Table 2. Comorbidity-PGS for age at the beginning of the 20% top distribution in individuals with PD from UK Biobank.

	OR	95% CI	p-value	Case/control High PGS	Case/Control Low PGS
**T2D-PGS**					
**<50**	5.76	0.93 - 35.7	0.0598	4/16	4/75
**50-70**	1.98	1.43 - 2.73	3.9×10^{-05}	65/249	150/1,107
**>70**	2.25	1.77 - 2.86	3.43×10^{-11}	133/338	284/1,597
**MDD-PGS**					
**<50**	1.25	0.345 - 4.54	0.734	6/14	17/62
**50-70**	1.74	1.3 - 2.32	2.22×10^{-04}	84/230	222/1,035
**>70**	1.10	0.844 - 1.43	0.483	87/384	321/1,560
**MH-PGS**					
**<50**	0.44	0.0253 - 7.47	0.566	1/19	3/76
**50-70**	1.95	0.905 - 4.18	0.0883	11/303	20/1,237
**>70**	1.60	0.83 - 3.09	0.16	13/458	32/1,849
**EPI-PGS**					
**<50**	2.84	0.342 - 23.6	0.334	2/18	3/76
**50-70**	2.21	1.33 - 3.67	2.32×10^{-03}	26/288	49/1,208
**>70**	1.24	0.778 - 1.97	0.366	26/445	82/1,799

Supplementary Table 3. Comorbidity-PGS for age at recruitment of the 20% top distribution in General population from UK Biobank.

	OR	95% CI	p-value	Case/control<br>High PGS	Case/Control<br>Low PGS
**T2D-PGS**					
**<50**	2.25	2.07 - 2.45	4.8310^{-79}	895/14,247	1,693/58,885
**50-70**	2.18	2.12 - 2.24	0	8,860/50,422	18,111/219,029
**>70**	1.91	1.44 - 2.54	8.52×10^{-06}	84/340	195/1,500
**MDD-PGS**					
**<50**	1.25	1.16 - 1.34	1.04×10^{-09}	1,062/14,082	3,499/57,077
**50-70**	1.24	1.19 - 1.28	2.42×10^{-30}	4,150/54,997	13,833/223,442
**>70**	1.23	0.808 - 1.86	0.338	31/393	110/1,585
**MH-PGS**					
**<50**	1.31	1.16 - 1.49	2.15×10^{-05}	332/14,812	1,030/59,546
**50-70**	1.28	1.2 - 1.38	3.23×10^{-12}	1,053/58,232	3286/233,851
**>70**	2.43	1.14 - 5.19	0.0221	11/413	19/1,676
**EPI-PGS**					
**<50**	1.15	0.995 - 1.33	0.0591	254/14,890	926/59,650
**50-70**	1.24	1.15 - 1.32	1.58×10^{-09}	1,133/58,152	3,771/233,366
**>70**	1.65	0.86 - 3.16	0.132	14/410	32/1,663

Supplementary Table 4. Comorbidity-PGS stratified by sex for the 20% top distribution in individuals with PD from UK Biobank.

	OR	95% CI	p-value	Case/control High PGS	Case/Control Low PGS
**T2D-PGS**					
**Female**	1.51	1.06 - 2.16	0.0239	49/243	137/1,028
**Male**	2.69	2.14 - 3.38	2.11×10^{-17}	155/338	291/1,682
**MDD-PGS**					
**Female**	1.47	1.09 - 1.98	0.0119	78/214	235/930
**Male**	1.21	0.933 - 1.57	0.151	91/401	310/1,664
**MH-PGS**					
**Female**	2.13	1.15 - 3.93	0.0155	17/275	31/1,134
**Male**	1.10	0.441 - 2.73	0.843	6/487	22/1,951
**EPI-PGS**					
**Female**	1.45	0.802 - 2.62	0.219	17/275	46/1,119
**Male**	1.51	0.99 - 2.31	0.0554	33/460	87/1,886

References

1. Mahajan A, Spracklen CN, Zhang W, et al. Multi-ancestry genetic study of type 2 diabetes highlights the power of diverse populations for discovery and translation. *Nat Genet*. 2022;54(5):560-572. doi:10.1038/s41588-022-01058-3

2. Howard DM, Adams MJ, Clarke TK, et al. Genome-wide meta-analysis of depression identifies 102 independent variants and highlights the importance of the prefrontal brain regions. *Nat Neurosci*. 2019;22(3):343-352. doi:10.1038/s41593-018-0326-7

3. Gormley P, Anttila V, Winsvold BS, et al. Meta-analysis of 375,000 individuals identifies 38 susceptibility loci for migraine. *Nature genetics*. 2016;48(8). doi:10.1038/ng.3598

4. Stevelink R, Campbell C, Chen S, et al. GWAS meta-analysis of over 29,000 people with epilepsy identifies 26 risk loci and subtype-specific genetic architecture. *Nat Genet*. 2023;55(9):1471-1482. doi:10.1038/s41588-023-01485-w

5. Barban N, Jansen R, de Vlaming R, et al. Genome-wide analysis identifies 12 loci influencing human reproductive behavior. *Nat Genet*. 2016;48(12):1462-1472. doi:10.1038/ng.3698
